# (2,2′-Bipyrimidine-κ^2^ *N* ^1^,*N* ^1′^)bis­(thio­cyanato-κ*N*)platinum(II)

**DOI:** 10.1107/S1600536812017552

**Published:** 2012-04-25

**Authors:** Kwang Ha

**Affiliations:** a School of Applied Chemical Engineering, The Research Institute of Catalysis, Chonnam National University, Gwangju 500-757, Republic of Korea

## Abstract

In the title complex, [Pt(NCS)_2_(C_8_H_6_N_4_)], the Pt^II^ ion is four-coordinated in a distorted square-planar environment defined by two pyrimidine N atoms derived from a chelating 2,2′-bipyrimidine (bpym) ligand and two mutually *cis* N atoms from two SCN^−^ anions. The thio­cyanate ligands are almost linear, displaying N—C—S bond angles of 178.6 (11) and 173.7 (11)°, and the N atoms are slightly bent coordinated to the Pt atom with Pt—N—C bond angles of 172.7 (9) and 160.4 (10)°. In the crystal, mol­ecules are held together by C—H⋯S hydrogen bonds. Intra­molecular C—H⋯N hydrogen bonds are also observed

## Related literature

For the crystal structures of related Pt^II^ complexes [Pt*X*_2_(bpym)] (*X* = Cl, I or Br), see: Kaim *et al.* (2002 ▶); Ha (2010 ▶, 2011 ▶).
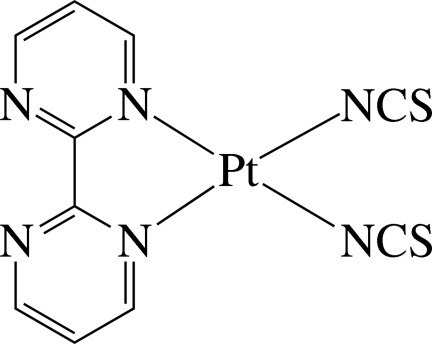


## Experimental

### 

#### Crystal data

[Pt(NCS)_2_(C_8_H_6_N_4_)]
*M* *_r_* = 469.42
Monoclinic, 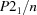

*a* = 11.0871 (8) Å
*b* = 9.8779 (7) Å
*c* = 12.8790 (9) Å
β = 115.135 (1)°
*V* = 1276.91 (16) Å^3^
*Z* = 4
Mo *K*α radiation
μ = 11.31 mm^−1^
*T* = 200 K
0.34 × 0.28 × 0.28 mm

#### Data collection

Bruker SMART 1000 CCD diffractometer
Absorption correction: multi-scan (*SADABS*; Bruker, 2000 ▶) *T*_min_ = 0.680, *T*_max_ = 1.000
7611 measured reflections
2467 independent reflections
2179 reflections with *I* > 2σ(*I*)
*R*_int_ = 0.026

#### Refinement

*R*[*F* ^2^ > 2σ(*F* ^2^)] = 0.043
*wR*(*F* ^2^) = 0.105
*S* = 1.09
2467 reflections
172 parameters
H-atom parameters constrained
Δρ_max_ = 4.86 e Å^−3^
Δρ_min_ = −1.72 e Å^−3^

### 

Data collection: *SMART* (Bruker, 2000 ▶); cell refinement: *SAINT* (Bruker, 2000 ▶); data reduction: *SAINT*; program(s) used to solve structure: *SHELXS97* (Sheldrick, 2008 ▶); program(s) used to refine structure: *SHELXL97* (Sheldrick, 2008 ▶); molecular graphics: *ORTEP-3* (Farrugia, 1997 ▶) and *PLATON* (Spek, 2009 ▶); software used to prepare material for publication: *SHELXL97*.

## Supplementary Material

Crystal structure: contains datablock(s) global, I. DOI: 10.1107/S1600536812017552/bt5882sup1.cif

Structure factors: contains datablock(s) I. DOI: 10.1107/S1600536812017552/bt5882Isup2.hkl

Additional supplementary materials:  crystallographic information; 3D view; checkCIF report

## Figures and Tables

**Table 1:** Selected bond lengths (Å)

Pt1—N1	2.014 (9)
Pt1—N4	1.999 (8)
Pt1—N5	1.958 (9)
Pt1—N6	2.017 (11)

**Table 2:** Hydrogen-bond geometry (Å, °)

*D*—H⋯*A*	*D*—H	H⋯*A*	*D*⋯*A*	*D*—H⋯*A*
C1—H1⋯N5	0.95	2.55	3.053 (15)	114
C8—H8⋯N6	0.95	2.62	3.138 (15)	115
C8—H8⋯S2^i^	0.95	2.87	3.496 (11)	124

Symmetry code: (i) 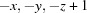.

**Fig. 1.**
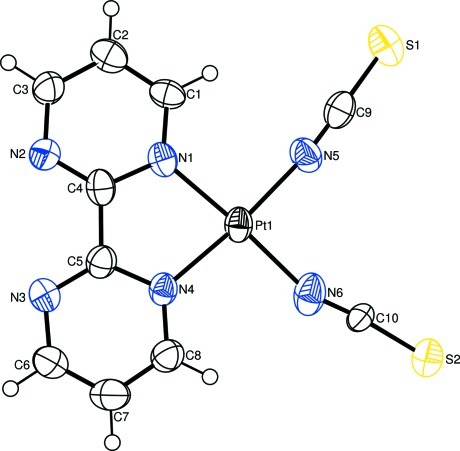
The molecular structure of the title complex, with atom numbering. Displacement ellipsoids are drawn at the 50% probability level for non-H atoms.

**Fig. 2.**
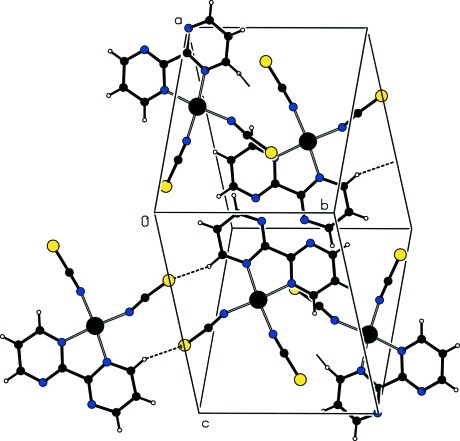
A view along the a axis of the crystal packing of the title complex. Intermolecular C—H···S hydrogen-bonds are shown as dashed lines (see Table 2 for details).

## supplementary crystallographic information

### Comment

Crystal structures of Pt^II^ complexes with 2,2'-bipyrimidine (bpym; C_8_H_6_N_4_) and halogen ions, [Pt*X*_2_(bpym)] (*X* = Cl, I or Br), have been reported previously (Kaim *et al.*, 2002; Ha, 2010; Ha, 2011).

In the title complex, [Pt(NCS)_2_(bpym)], the Pt^II^ ion is four-coordinated in a distorted square-planar environment defined by two pyrimidine N atoms derived from a chelating bpym ligand and two mutually *cis* N atoms from two SCN^-^ anions (Fig. 1). The main contribution to the distortion is the tight N1—Pt1—N4 chelate angle of 80.9 (3)°, which results in non-linear *trans* axes [<N1—Pt1—N6 = 176.2 (4)° and <N4—Pt1—N5 = 174.5 (4)°]. The Pt—N(bpym) and Pt—N(NCS) bond lengths are nearly equivalent [Pt—N: 1.958 (9)–2.017 (11) Å] (Table 1). The thiocyanato ligands are almost linear displaying N—C—S bond angles of 178.6 (11)° and 173.7 (11)°, and the N atoms are slightly bent coordinated to the Pt atom with the Pt—N—C bond angles of 172.7 (9)° and 160.4 (10)°, characteristic of an N-bonded conformation. The nearly planar bpym ligand [maximum deviation = 0.09 (1) Å] is slightly inclined to the least-squares plane of the PtN_4_ unit [maximum deviation = 0.015 (5) Å], making a dihedral angle of 3.6 (5)°. In the crystal, two complex molecules are assembled by intermolecular C—H···S hydrogen bonds with C···S = 3.496 (11) Å, forming a dimer-type species (Fig. 2, Table 2). Intramolecular C—H···N hydrogen bonds are also observed (Table 2).

### Experimental

To a solution of K_2_PtCl_4_ (0.2087 g, 0.503 mmol) in H_2_O (15 ml) and acetone (15 ml) were added KSCN (0.5071 g, 5.218 mmol) and 2,2'-bipyrimidine (0.0809 g, 0.512 mmol), and refluxed for 3 h. After evaporation of the solvent, the residue was dissolved in CH_3_CN (20 ml), then filtered through a plug of silica gel (2 cm *x* 7 cm). The solvent of the eluate was removed *in vacuo*, the residue was washed with ether, and dried at 323 K, to give an orange powder (0.0686 g). Orange block-like crystals, suitable for X-ray analysis, were obtained by slow evaporation of a CH_3_CN solution at room temperature.

### Refinement

H atoms were included in calculated positions and treated as riding atoms: C—H = 0.95 Å with *U*_iso_(H) = 1.2*U*_eq_(C). The highest peak (4.86 e Å^-3^) and the deepest hole (-1.72 e Å^-3^) in the difference Fourier map are located 0.91 Å and 0.79 Å, respectively, from the Pt1 atom.

### Crystal data

**Table:**

[Pt(NCS)_2_(C_8_H_6_N_4_)]	*F*(000) = 872
*M**_r_* = 469.42	*D*_x_ = 2.442 Mg m^−^^3^
Monoclinic, *P*2_1_/*n*	Mo *K*α radiation, λ = 0.71073 Å
Hall symbol: -P 2yn	Cell parameters from 5056 reflections
*a* = 11.0871 (8) Å	θ = 2.7–26.0°
*b* = 9.8779 (7) Å	µ = 11.31 mm^−^^1^
*c* = 12.8790 (9) Å	*T* = 200 K
β = 115.135 (1)°	Block, orange
*V* = 1276.91 (16) Å^3^	0.34 × 0.28 × 0.28 mm
*Z* = 4	

### Data collection

**Table:**

Bruker SMART 1000 CCD diffractometer	2467 independent reflections
Radiation source: fine-focus sealed tube	2179 reflections with *I* > 2σ(*I*)
Graphite monochromator	*R*_int_ = 0.026
φ and ω scans	θ_max_ = 26.0°, θ_min_ = 2.0°
Absorption correction: multi-scan (*SADABS*; Bruker, 2000)	*h* = −13→13
*T*_min_ = 0.680, *T*_max_ = 1.000	*k* = −12→11
7611 measured reflections	*l* = −15→12

### Refinement

**Table:**

Refinement on *F*^2^	Primary atom site location: structure-invariant direct methods
Least-squares matrix: full	Secondary atom site location: difference Fourier map
*R*[*F*^2^ > 2σ(*F*^2^)] = 0.043	Hydrogen site location: inferred from neighbouring sites
*wR*(*F*^2^) = 0.105	H-atom parameters constrained
*S* = 1.09	*w* = 1/[σ^2^(*F*_o_^2^) + (0.030*P*)^2^ + 21.4936*P*] where *P* = (*F*_o_^2^ + 2*F*_c_^2^)/3
2467 reflections	(Δ/σ)_max_ < 0.001
172 parameters	Δρ_max_ = 4.86 e Å^−^^3^
0 restraints	Δρ_min_ = −1.72 e Å^−^^3^

### Special details

**Table:**

Geometry. All e.s.d.'s (except the e.s.d. in the dihedral angle between two l.s. planes) are estimated using the full covariance matrix. The cell e.s.d.'s are taken into account individually in the estimation of e.s.d.'s in distances, angles and torsion angles; correlations between e.s.d.'s in cell parameters are only used when they are defined by crystal symmetry. An approximate (isotropic) treatment of cell e.s.d.'s is used for estimating e.s.d.'s involving l.s. planes.
Refinement. Refinement of *F*^2^ against ALL reflections. The weighted *R*-factor *wR* and goodness of fit *S* are based on *F*^2^, conventional *R*-factors *R* are based on *F*, with *F* set to zero for negative *F*^2^. The threshold expression of *F*^2^ > σ(*F*^2^) is used only for calculating *R*-factors(gt) *etc*. and is not relevant to the choice of reflections for refinement. *R*-factors based on *F*^2^ are statistically about twice as large as those based on *F*, and *R*- factors based on ALL data will be even larger.

### Fractional atomic coordinates and isotropic or equivalent isotropic displacement parameters (Å^2^)

**Table:**

	*x*	*y*	*z*	*U*_iso_*/*U*_eq_	
Pt1	0.16191 (4)	0.40380 (4)	0.58301 (3)	0.04005 (15)	
S1	−0.0058 (3)	0.6087 (4)	0.8261 (3)	0.0601 (8)	
S2	0.0260 (3)	−0.0068 (3)	0.6854 (3)	0.0519 (7)	
N1	0.2144 (8)	0.5882 (9)	0.5497 (7)	0.0382 (18)	
N2	0.3270 (10)	0.6962 (9)	0.4524 (8)	0.050 (2)	
N3	0.3600 (10)	0.4377 (10)	0.3743 (8)	0.049 (2)	
N4	0.2425 (8)	0.3496 (9)	0.4767 (7)	0.0385 (18)	
N5	0.0853 (9)	0.4748 (10)	0.6838 (8)	0.048 (2)	
N6	0.1173 (10)	0.2136 (11)	0.6120 (8)	0.058 (3)	
C1	0.1920 (10)	0.7047 (11)	0.5879 (8)	0.042 (2)	
H1	0.1456	0.7078	0.6352	0.051*	
C2	0.2375 (11)	0.8237 (12)	0.5579 (9)	0.048 (3)	
H2	0.2214	0.9091	0.5835	0.057*	
C3	0.3061 (12)	0.8148 (11)	0.4906 (9)	0.049 (3)	
H3	0.3390	0.8950	0.4711	0.059*	
C4	0.2822 (10)	0.5887 (11)	0.4827 (8)	0.041 (2)	
C5	0.2989 (10)	0.4514 (11)	0.4419 (9)	0.041 (2)	
C6	0.3601 (12)	0.3115 (12)	0.3349 (10)	0.054 (3)	
H6	0.4017	0.2973	0.2848	0.064*	
C7	0.3040 (13)	0.2034 (12)	0.3627 (10)	0.055 (3)	
H7	0.3042	0.1161	0.3317	0.066*	
C8	0.2465 (11)	0.2250 (11)	0.4376 (9)	0.047 (2)	
H8	0.2097	0.1511	0.4615	0.056*	
C9	0.0462 (10)	0.5294 (12)	0.7422 (9)	0.046 (3)	
C10	0.0757 (10)	0.1268 (10)	0.6445 (9)	0.038 (2)	

### Atomic displacement parameters (Å^2^)

**Table:**

	*U*^11^	*U*^22^	*U*^33^	*U*^12^	*U*^13^	*U*^23^
Pt1	0.0392 (2)	0.0493 (3)	0.0308 (2)	−0.00470 (17)	0.01392 (16)	0.00473 (17)
S1	0.0539 (17)	0.082 (2)	0.0512 (17)	0.0017 (16)	0.0284 (14)	−0.0038 (16)
S2	0.0481 (15)	0.0567 (17)	0.0519 (16)	−0.0078 (13)	0.0222 (13)	0.0038 (13)
N1	0.035 (4)	0.052 (5)	0.025 (4)	0.000 (4)	0.010 (3)	0.002 (4)
N2	0.068 (6)	0.040 (5)	0.051 (5)	−0.003 (4)	0.034 (5)	0.000 (4)
N3	0.059 (6)	0.050 (6)	0.047 (5)	−0.002 (4)	0.030 (5)	−0.001 (4)
N4	0.041 (4)	0.043 (5)	0.030 (4)	−0.005 (4)	0.014 (4)	0.002 (4)
N5	0.052 (5)	0.058 (6)	0.040 (5)	−0.004 (5)	0.025 (4)	−0.007 (4)
N6	0.062 (6)	0.072 (7)	0.039 (5)	−0.018 (5)	0.020 (5)	0.003 (5)
C1	0.046 (6)	0.045 (6)	0.032 (5)	0.003 (5)	0.013 (4)	−0.009 (4)
C2	0.049 (6)	0.056 (7)	0.032 (5)	0.002 (5)	0.012 (5)	−0.008 (5)
C3	0.062 (7)	0.043 (6)	0.047 (6)	−0.012 (5)	0.028 (5)	−0.007 (5)
C4	0.036 (5)	0.055 (6)	0.029 (5)	−0.003 (5)	0.011 (4)	0.004 (4)
C5	0.040 (5)	0.043 (6)	0.040 (6)	−0.005 (4)	0.016 (5)	0.000 (4)
C6	0.066 (7)	0.050 (7)	0.052 (7)	0.003 (6)	0.032 (6)	−0.005 (5)
C7	0.072 (8)	0.044 (6)	0.047 (6)	0.008 (6)	0.021 (6)	−0.002 (5)
C8	0.053 (6)	0.043 (6)	0.043 (6)	−0.007 (5)	0.019 (5)	−0.002 (5)
C9	0.043 (6)	0.048 (6)	0.041 (6)	−0.006 (5)	0.011 (5)	0.009 (5)
C10	0.048 (6)	0.028 (5)	0.041 (5)	−0.006 (4)	0.023 (5)	0.001 (4)

### Geometric parameters (Å, º)

**Table:**

Pt1—N1	2.014 (9)	N5—C9	1.151 (14)
Pt1—N4	1.999 (8)	N6—C10	1.135 (13)
Pt1—N5	1.958 (9)	C1—C2	1.397 (16)
Pt1—N6	2.017 (11)	C1—H1	0.9500
S1—C9	1.625 (13)	C2—C3	1.378 (15)
S2—C10	1.602 (10)	C2—H2	0.9500
N1—C1	1.316 (13)	C3—H3	0.9500
N1—C4	1.363 (13)	C4—C5	1.494 (15)
N2—C4	1.301 (13)	C6—C7	1.359 (17)
N2—C3	1.328 (14)	C6—H6	0.9500
N3—C5	1.318 (14)	C7—C8	1.380 (16)
N3—C6	1.346 (14)	C7—H7	0.9500
N4—C8	1.338 (13)	C8—H8	0.9500
N4—C5	1.357 (13)		
			
N5—Pt1—N4	174.5 (4)	C1—C2—H2	120.6
N5—Pt1—N1	93.6 (4)	N2—C3—C2	121.3 (11)
N4—Pt1—N1	80.9 (3)	N2—C3—H3	119.4
N5—Pt1—N6	90.1 (4)	C2—C3—H3	119.4
N4—Pt1—N6	95.4 (4)	N2—C4—N1	125.1 (10)
N1—Pt1—N6	176.2 (4)	N2—C4—C5	121.0 (9)
C1—N1—C4	118.5 (9)	N1—C4—C5	113.8 (9)
C1—N1—Pt1	126.4 (7)	N3—C5—N4	125.5 (10)
C4—N1—Pt1	115.1 (7)	N3—C5—C4	120.1 (9)
C4—N2—C3	117.4 (9)	N4—C5—C4	114.4 (9)
C5—N3—C6	115.3 (10)	N3—C6—C7	123.7 (11)
C8—N4—C5	117.6 (9)	N3—C6—H6	118.1
C8—N4—Pt1	126.9 (7)	C7—C6—H6	118.1
C5—N4—Pt1	115.5 (7)	C6—C7—C8	117.5 (11)
C9—N5—Pt1	172.7 (9)	C6—C7—H7	121.3
C10—N6—Pt1	160.4 (10)	C8—C7—H7	121.3
N1—C1—C2	118.9 (10)	N4—C8—C7	120.3 (10)
N1—C1—H1	120.5	N4—C8—H8	119.8
C2—C1—H1	120.5	C7—C8—H8	119.8
C3—C2—C1	118.8 (10)	N5—C9—S1	178.6 (11)
C3—C2—H2	120.6	N6—C10—S2	173.7 (11)
			
N5—Pt1—N1—C1	−1.9 (8)	Pt1—N1—C4—N2	−178.9 (8)
N4—Pt1—N1—C1	177.6 (9)	C1—N1—C4—C5	−178.5 (8)
N5—Pt1—N1—C4	176.6 (7)	Pt1—N1—C4—C5	2.8 (10)
N4—Pt1—N1—C4	−3.9 (7)	C6—N3—C5—N4	2.6 (16)
N1—Pt1—N4—C8	−175.8 (9)	C6—N3—C5—C4	−174.8 (10)
N6—Pt1—N4—C8	5.2 (9)	C8—N4—C5—N3	−1.5 (16)
N1—Pt1—N4—C5	4.4 (7)	Pt1—N4—C5—N3	178.3 (9)
N6—Pt1—N4—C5	−174.5 (7)	C8—N4—C5—C4	176.0 (9)
N5—Pt1—N6—C10	5 (3)	Pt1—N4—C5—C4	−4.2 (11)
N4—Pt1—N6—C10	−174 (3)	N2—C4—C5—N3	0.2 (16)
C4—N1—C1—C2	0.4 (14)	N1—C4—C5—N3	178.5 (9)
Pt1—N1—C1—C2	178.9 (7)	N2—C4—C5—N4	−177.5 (9)
N1—C1—C2—C3	−0.9 (15)	N1—C4—C5—N4	0.9 (13)
C4—N2—C3—C2	−1.2 (17)	C5—N3—C6—C7	−1.1 (18)
C1—C2—C3—N2	1.3 (17)	N3—C6—C7—C8	−1.4 (19)
C3—N2—C4—N1	0.7 (16)	C5—N4—C8—C7	−1.3 (15)
C3—N2—C4—C5	178.8 (10)	Pt1—N4—C8—C7	178.9 (8)
C1—N1—C4—N2	−0.3 (15)	C6—C7—C8—N4	2.6 (17)

### Hydrogen-bond geometry (Å, º)

**Table:**

*D*—H···*A*	*D*—H	H···*A*	*D*···*A*	*D*—H···*A*
C1—H1···N5	0.95	2.55	3.053 (15)	114
C8—H8···N6	0.95	2.62	3.138 (15)	115
C8—H8···S2^i^	0.95	2.87	3.496 (11)	124

Symmetry code: (i) −*x*, −*y*, −*z*+1.
